# Analysis of miRNA Profiles and the Regulatory Network in Congenital Pulmonary Airway Malformations

**DOI:** 10.3389/fped.2021.671107

**Published:** 2021-11-18

**Authors:** Jiahang Zeng, Wei Liu, Jianhua Liang, Junzheng Peng, Fenghua Wang, Jue Tang, Qinglin Yang, Linwei Zhuang, Dongmei Huang, Le Li

**Affiliations:** Department of Thoracic Surgery, Guangzhou Women and Children's Medical Center, Guangzhou Medical University, Guangzhou, China

**Keywords:** miRNA, congenital pulmonary airway malformations, biomarker, miRNA chip, type I and type II CPAM

## Abstract

**Background:** Specific diagnostic markers for congenital pulmonary airway malformations (CPAMs) have not yet been discovered. This study intends to detect differentially expressed miRNAs in type I and type II CPAMs by using a miRNA chip and clarify the feasibility of miRNAs as different CPAM typing markers.

**Methods:** Lung tissues of type I and type II CPAMs were collected and used to assess the differentially expressed miRNAs using a miRNA chip after evaluation using hematoxylin–eosin staining and Masson staining. Quantitative reverse transcription-polymerase chain reaction and fluorescence in situ hybridization were used to verify the quality of the miRNA chip. The function and pathways of related differentially expressed miRNAs were analyzed by Gene Ontology Enrichment (GO) analysis and Kyoto Encyclopedia of Genes and Genomes (KEGG) analysis, respectively. Targets of miRNAs were predicted by targetscan7.1 and mirdbV6 and the network between miRNA and mRNA was established using Cystoscope software.

**Results:** In total, 394/34 upregulated and 321/72 downregulated miRNAs were found in type I and type II CPAMs, respectively. GO and KEGG analysis showed that different pathways are involved in the regulation of CPAM, including platelet activation, Ras, MAPK, FoxO, and PI3K-Akt signaling pathways. miRNA–mRNA network analysis confirmed four major miRNAs in CPAM, including miR-4731-5p to complexin 2, miR-3150a-3p to vesicle amine transport 1, miR-32-5p to F-box and WD repeat domain containing 7, and miR-454-3p to SLAIN motif family member 1.

**Conclusion:** In summary, we have identified four candidate miRNAs and pathways related to different pattern CPAMs, which provide a new perspective for CPAM research and treatment.

## Introduction

Congenital pulmonary airway malformation (CPAM) is a clinically rare congenital malformation of the lung, characterized by localized pulmonary dysplasia or abnormality that accounts for ~20–30% of congenital lung malformations (1). CPAM is divided into five types according to histological criteria, including type 0 to V (2). The current diagnosis is mainly based on imaging and histopathological biopsy. However, its clinical manifestations are diverse, easily missed, and misdiagnosed, and it can progress to malignancy (3, 4). Therefore, it is of great significance to accurately diagnose fetal CPAM to improve pregnancy outcomes, including its type, lesion range, and complications. Although an increasing number of studies have been focused on the qualitative, pathophysiology, prenatal ultrasound diagnosis, and various conservative or surgical treatments of CPAM, there is still a lack of comprehensive understanding of CPAM, which limits the clinical treatment to CPAM. Therefore, it is crucial to delve into the mechanism of CPAM to develop new CPAM classification, diagnosis, and treatment methods.

The pathogenesis of CPAM is very complicated and unclear. The causes of CPAM mainly are excessive interstitial hyperplasia caused by bronchial atresia and the imbalance of cell proliferation and apoptosis during lung development (5, 6). Furthermore, several key factors were also investigated, including SOX2, SOX9, and FGF signaling pathways (7–9). However, the mechanism of CPAM remains far from fully elaborated. MicroRNA is a class of noncoding RNA molecules with a highly conserved structure that can bind to the 3′ UTR region of a target messenger to block translation of target mRNA or induce the degradation of target mRNA (10, 11). It has been demonstrated that miRNAs are widely involved in various biological processes, including gene regulation, cell proliferation, differentiation, and apoptosis, and tissue and organ morphogenesis (12–14). With the continuous deepening research on miRNA, increasing numbers of studies indicated the epigenetic regulation of miRNAs on lung development and lung diseases involving various physiological and pathological processes of the lungs. First, miRNAs play an important role in cell proliferation and differentiation of lung tissue. For example, the miR-17-92 family can regulate the survival and proliferation of cells in the early and late stages of lung development (15), whereas the miR-200 family and its targets regulate the differentiation of type II cells in the human fetal lung (16). Second, miRNAs play an important role in lung diseases *via* regulating different processes. Let-7d has been identified as a key miRNA involved in the pathogenesis of idiopathic pulmonary fibrosis (17). MiR-126 is reported to play a key role in the development of allergic airway disease (18). Third, miRNAs can be used as biomarkers in lung development and lung diseases, such as miR-520f, plasma miRNA-23a, miRNA-451, and miRNA-17 (19, 20). In addition, miRNAs play an important role in lung development and diseases by regulating target genes or participating in the regulation of signaling pathways. For example, microRNA-1 regulates chondrocyte phenotype by inhibiting histone deacetylase 4 during growth plate development (20). Furthermore, miR-128 directly targets vascular endothelial growth factor-C in human non-small cell lung cancer tumorigenesis, angiogenesis, and lymphangiogenesis (21).

The above studies suggest that miRNA and its regulated target mRNA play an important role in regulating lung development and the pathogenesis of lung diseases. However, the expression and role of miRNAs and mRNAs in CPAM have rarely been reported. Therefore, this study intends to mine the differentially expressed miRNAs in type I and type II CPAMs by using miRNA chips and clarify the feasibility of miRNAs as different CPAM typing markers by investigating the pathways of miRNA involved. This study not only has important value for the diagnosis and prognosis of CPAM but also provides a basis for evaluating the outcome of fetal pregnancy.

## Materials and Methods

### Sample Collection and RNA Extraction

Lung tissue specimens were collected from Guangzhou Women and Children's Medical Center, including three lung tissues from type I CAPM patients, three lung tissues from type II CAPM patients, the adjacent tissues as control (*N* = 3). All participants signed an informed consent form. All experiments were approved by the Medical Ethics Committee of Guangzhou Women and Children's Medical Center, Guangzhou Medical University. The RNA was isolated using the RNeasy Mini Kit (74104, QIAGEN). NanoDrop ND-1000 was used to measure the RNA quantity and quality and an A260/A280 ratio close to 2.0 was considered pure RNA. Standard denaturing agarose gel electrophoresis was used to assess RNA integrity. Sharp and intense bands of 28S and 18S ribosomal RNA represented the qualified RNA integrity, and the intensity ratio of the 28S band/18S bands was ~2-fold.

### RNA Labeling and Array Hybridization

RNA labeling and array hybridization was performed according to the protocol of Agilent miRNA Microarray System with miRNA Complete Labeling and Hyb Kit (Agilent Technology). Total miRNA from different samples was labeled by Cyanine 3-pCp and the labeled cRNA was redissolved in water after inspissation and desiccation. One micrograms of labeled cRNA was heated at 60°C for 30 min after fragmentation by 11 μL 10× blocking agent and 2.2 μL 25× fragmentation buffer. Finally, the labeled cRNA was diluted in 55 μL of 2× GE hybridization buffer. One hundred microliters of the hybridization solution was dispensed into the gasket slide and assembled to the gene expression microarray slide and then incubated for 17 h at 65°C in an Agilent Hybridization Oven. The Agilent Microarray Scanner (part number G2505C) was used to scan the hybridized arrays after washing and fixing.

### Data Processing and Analysis

Array images were analyzed using Agilent Feature Extraction software (version 11.0.1.1). The GeneSpring GX v14.9 software package (Agilent Technologies) was used to normalize quantile and process subsequent data acquired from the miRNA chip. Processed data were verified and optimized by alignment to human miRNA transcriptome, which represented all known miRNAs. miRNAs that at least three out of nine samples have flags in detected were chosen for subsequently analysis after normalization. Differentially expressed miRNAs between the two samples were filtered using the characteristic log 2 (Fold Change) ≥ 1.0, *P* ≤ 0.05, and FDR ≤ 1.0. Differentially expressed miRNAs with statistical significance between the two groups were presented through Volcano Plot and Hierarchical Clustering. R scripts were used to draw the hierarchical clustering.

### Gene Ontology Analysis and Kyoto Encyclopedia of Genes and Genomes Pathway Analysis

The targets from the GEO database were analyzed by an online program Database for Annotation, Visualization, and Integrated Discovery (DAVID) (https://david.ncifcrf.gov/) (22). The GO chord R package and DAVID database were used to perform GO analysis and KEGG pathway maps with cut-off *p* < 0.05, respectively.

### miRNA-mRNA Network Construction

miRNA's target genes were predicted using targetscan7.1 (http://www.targetscan.org/mmu_71/) and mirdbV6A (http://mirdb.org/miRDB/) databases. In addition, microRNA–target interactions were experimentally validated using the database mirTarbase7.0 (http://mirtarbase.mbc.nctu.edu.tw/php/index.php). The targets of miRNAs were identified by Cytoscape, and modules of key miRNAs were screened by the Molecular Complex Detection (MCODE) with the following default parameters: node score cut-off =0.2, cut-off =2, k-core = 2, and max depth = 100.

### Histopathological Staining

Lung tissues were fixed with 4% paraformaldehyde and embedded in paraffin which were sliced into 4 μm sections. For hematoxylin–eosin staining, the section was stained with hematoxylin for 5–8 min after dewaxing and then stained with eosin for 1–3 min. For Masson staining, sections were stained with celestine blue dye solution for 5 min, followed by staining with hematoxylin, ponceau acid fuchsin for 5–8 min, respectively. Then, sections were differentiated with 1% phosphomolybdic acid aqueous solution for 5–10 min and stained with 1% bright green dye for 2–5 min, and mounting. After dehydration, these tissue sections were analyzed under a microscope (Olympus, Japan).

### Quantitative Reverse Transcription-Polymerase Chain Reaction

TRIzol reagent (9109, Takara) was used to extract total RNA from lung tissues. Two micrograms of RNA was used for cDNA synthesis using BestarTM qPCR RT kit (2220, DBI) according to the manufacturer's instructions. qRT-PCR was performed using ABI 7500 instrument (ABI7500, ABI, Foster City, CA, USA) in a 20 μL reaction volume, which included 10 μL of Bestar® SybrGreen qPCR master Mix (2043, DBI), 0.5 μL of each primer (10 μM), 1 μL of the cDNA template, and 8 μL of ddH2O. Amplification processes were as follows: 95°C, 2 min; 94°C, 20 s and 58°C, 20 s and 72°C, 20 s for 40 cycles. The relative expression of miRNAs was normalized to U6. Relative expression levels were calculated by 2^−ΔΔCt^ methods. Primers were synthesized by Sangon Biotech, the details of which are listed in Table 1.

**Table 1:** Primers used in the present study.

**ID**	**Sequence (5′-3′)**
U6-F	CTCGCTTCGGCAGCACA
U6-R	AACGCTTCACGAATTTGCGT
All R	TCCACGACACCAGTTGAG
hsa-miR-590-3p	TAATTTTATGTATAAGCTAGT
hsa-miR-590-3p RT	CTCAACTGGTGTCGTGGAGTCGGCAATTCAGTT GAGACTAGCTT
hsa-miR-590-3p F	ACACTCCAGCTGGGTAATTTTATGTATAAGC
hsa-miR-146a-3p	CCTCTGAAATTCAGTTCTTCAG
hsa-miR-146a-3p RT	CTCAACTGGTGTCGTGGAGTCGGCAATTCAGTT GAGCTGAAGAA
hsa-miR-146a-3p F	ACACTCCAGCTGGGCCTCTGAAATTCAGTTCT
hsa-miR-let-7e-3p	CTATACGGCCTCCTAGCTTTCC
hsa-miR-let-7e-3p RT	CTCAACTGGTGTCGTGGAGTCGGCAATTCAGTT GAGGGAAAGCT
hsa-miR-let-7e-3p F	ACACTCCAGCTGGGCTATACGGCCTCCTAGCT
hsa-miR-21-3p	CAACACCAGTCGATGGGCTGT
hsa-miR-21-3p RT	ACACTCCAGCTGGGCAACACCAGTCGATGGGC
hsa-miR-21-3p F	CAACACCAGTCGATGGGCTGT
hsa-miR-140-5p	CAGTGGTTTTACCCTATGGTAG
hsa-miR-140-5p RT	GTCGTATCCAGTGCAGGGTCCGAGGTGCACTG GATACGACCTACCAT
hsa-miR-140-5p F	CAGTGGTTTTACCCTATGGTAG

### Fluorescence *in situ* Hybridization Analysis for miRNA

The distribution of miRNA was identified by FISH. Briefly, lung tissue was fixed by 4% paraformaldehyde and cut into 4 μm sections. Then, these sections were pre-hybridized in 200 μL of prehybridization solution for 1 h at 37°C and then hybridized with 250 μL of hybridization solution containing FAM probe (6 ng/μl) overnight at 37°C. Mouse anti-DIG-HRP (G1401, Servicebio) and CY3-TSA (G3016-3, Servicebio) were added to the slides and incubated in a dark condition for 5 min after washing off the hybridization. Cells were then stained with DAPI (1:800) and incubated for 8 min in the dark after washing with wash buffer at 42°C. Finally, different fields were observed under a fluorescence microscope (Olympus, Japan) with FAM at 488 nm. The sequences of the probes are as follows: miR-590-3p: 5′-ACTAGCTTATACATAAAATTA-3′; miR-146a-3p: 5′-CTGAAGAACTGAATTTCAGAGG-3′; miR-140-5p: 5′-CTGTTTGTGGCTGGGCAGACGA-3′; miR-Let-7e-3p: 5′-GGAAAGCTAGGAGGCCGTATAG-3′; miR-21-3p: 5′-CTACCATCGTGACATCTCCATG-3′.

### Dual-Luciferase Reporter Assay

To binding sites of miR-4731-5p for complexin 2 (CPLX2), miR-3150a-3p to vesicle amine transport 1 (VAT1), miR-32-5p to F-box and WD repeat domain containing 7 (FBXW7), and miR-454-3p to SLAIN motif family member 1 (SLAIN1) were predicted using TargetScan. The 3′ UTR of CPLX2, VAT1, FBXW7, and SLAIN1 containing the seed binding site and its mutant sequences were synthesized and then cloned into the pmirGLO vector (Promega), respectively. 293T cells were cotransfected with the pmirGLO vector and miRNA mimics using the Lipo3000 Reagent (Invitrogen). Cell lysates were harvested at 48 h after transfection, and luciferase activities were determined using the Dual-Luciferase Reporter System (Promega) and a microplate reader (Tecan M1000, Switzerland). The sequence of miRNA mimics and miRNA NC were as follows: miR-4731-5p: XX, miR-3150a-3p: XX, miR-32-5p: XX, miR-454-3p: XX, miR-NC: sense: 5′-UUCUCCGAACGUGUCACGUTT-3′, antisense: 5′-ACGUGACACGUUCGGAGAATT-3′.

### Statistical Analysis

All statistical analyses were completed using SPSS 21.0 software (IBM, Armonk, NY, USA). The data were presented as mean ± standard deviation. At least three independent experiments were performed. Statistical comparisons were conducted using an unpaired *t*-test between two groups, and one-way analysis of variance (ANOVA) was used to compare more than two groups. The level of statistical significance was set at *p* < 0.05.

## Results

### microRNA Microarray Profiling and Differentially Expressed miRNAs Between Type I or Type II CPAM and the Control Group

**Figure 1:**
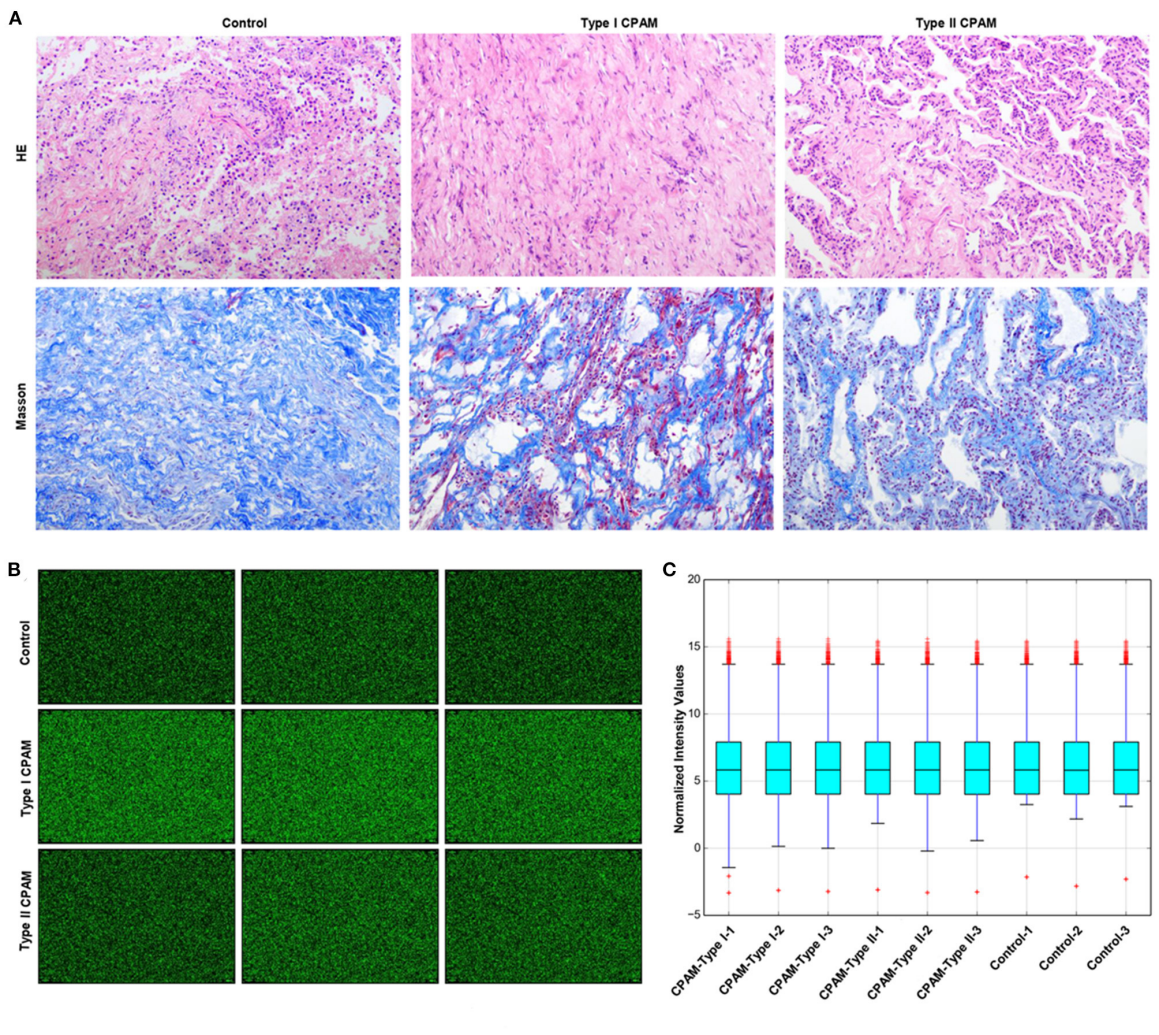
miRNA chip information of type I and type II CPAM detected using the Agilent 8 × 60 K miRNA-array platform. **(A)** HE and Masson staining analysis of lung tissues from type I and type II CPAM, **(B)** miRNA chip images scanned by Agilent Feature Extraction software (v11.0.1.1), and **(C)** Normalized intensity values of miRNA chip signals in type I, type II CPAM, and control groups.

**Figure 2:**
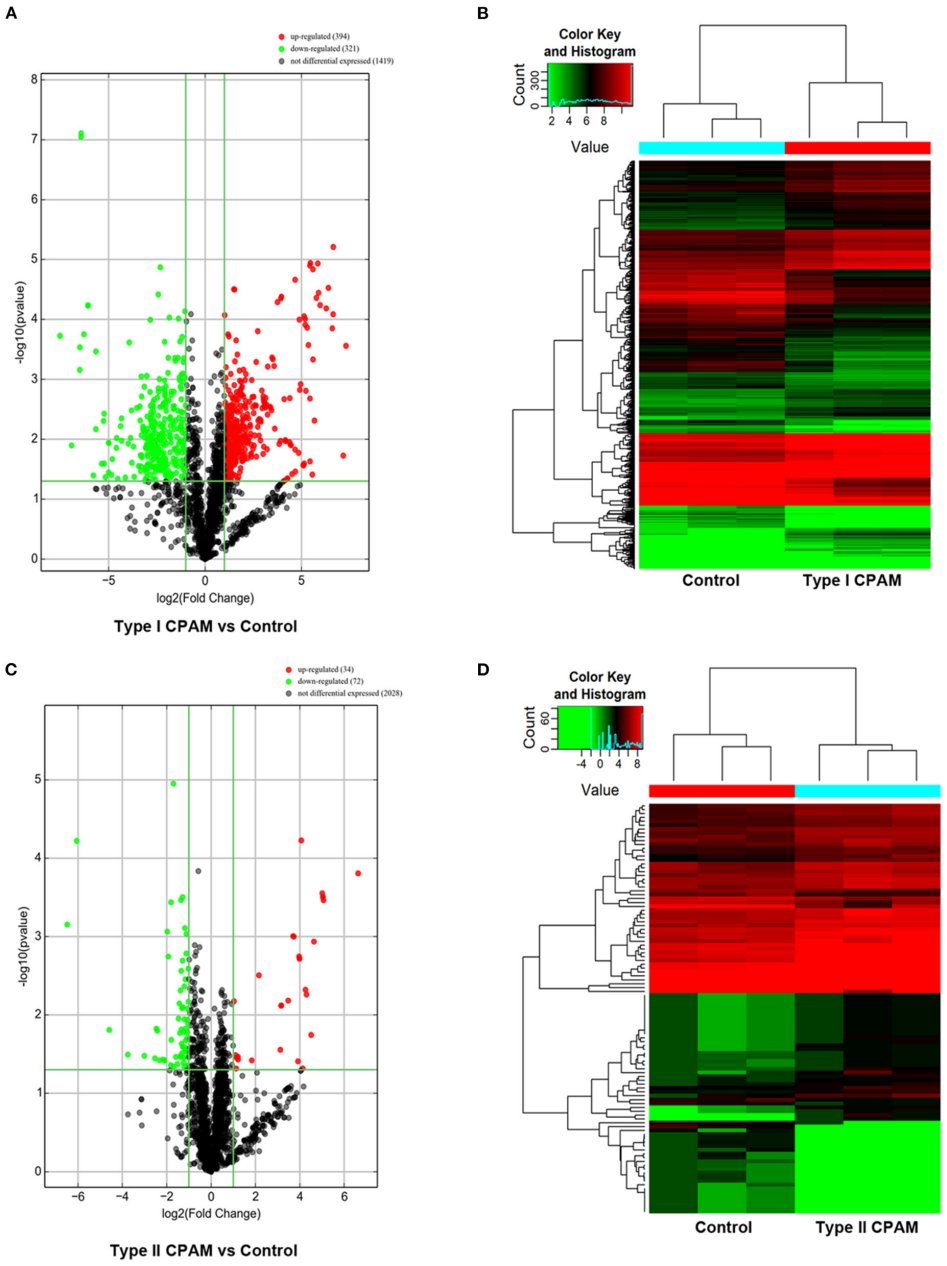
Differentially expressed miRNAs with statistical significance between type I, type II CPAM, and control groups. **(A,B)** Volcano plot of differentially expressed miRNAs between type I CPAM or type II CPAM groups and control group, **(C,D)** hierarchical clustering of the differentially expressed miRNAs between type I CPAM or type II CPAM groups and control group. The differentially expressed miRNAs were identified through fold change filtering ≥ 1.0 and false discovery rate ≤ 1.0 (*P* ≤ 0.05). Hierarchical clustering was performed using the R scripts.

**Figure 3:**
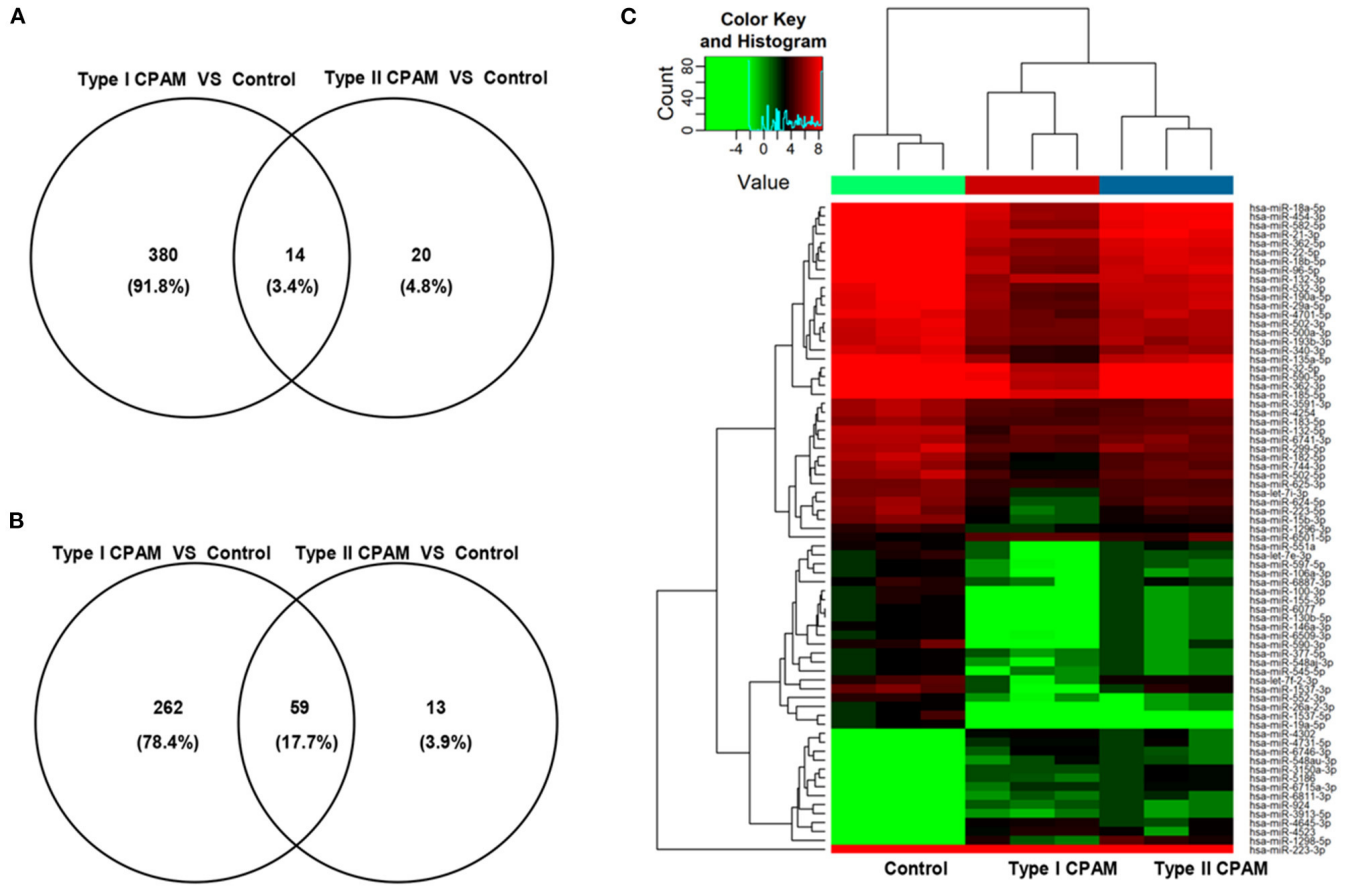
Common differentially expressed miRNAs between type I and type II CPAM compared with control groups. **(A,B)** Venn diagram of common up- and downregulated differentially expressed miRNAs between type I and type II CPAM compared with control groups and **(C)** Hierarchical clustering to present the common differentially expressed miRNAs between type I CPAM or type II CPAM groups and control group.

Type I and type II CPAM patients were collected to perform miRNA chip. As shown in Figure 1A, HE analysis showed that multiple cysts of different sizes were observed in both type I and type II CPAM. Of these, type I CPAM was lined with pseudostratified ciliated columnar epithelium along with mucous cells, whereas type II CPAM was lined with cuboidal to columnar epithelium. Masson staining showed that the collagen fibers were decreased but muscle fibers were increased in both type I and type II CPAM compared with control. Among the 2,549 miRNAs tested using the Agilent 8 × 60 K miRNA-array platform, a total of 2,134 miRNAs were found (Figures 1B,C and Supplementary Table 1). A total of 715 miRNAs were found to be differentially expressed with 394 upregulation and 321 downregulation in the type I CPAM patients (Figure 2A), and 106 miRNAs showed differential expression with 34 upregulation and 72 downregulation in the type II CPAM patients (Figure 2B) compared to healthy individuals (Supplementary Table 2). The results also were presented by heat map (Figures 2C,D). Further analysis showed that 14 upregulated (Figures 3A,C) and 59 downregulated (Figures 3B,C) miRNAs were found in both type I and type II CPAM patients.

### Validation of Selected miRNAs Expression

**Figure 4:**
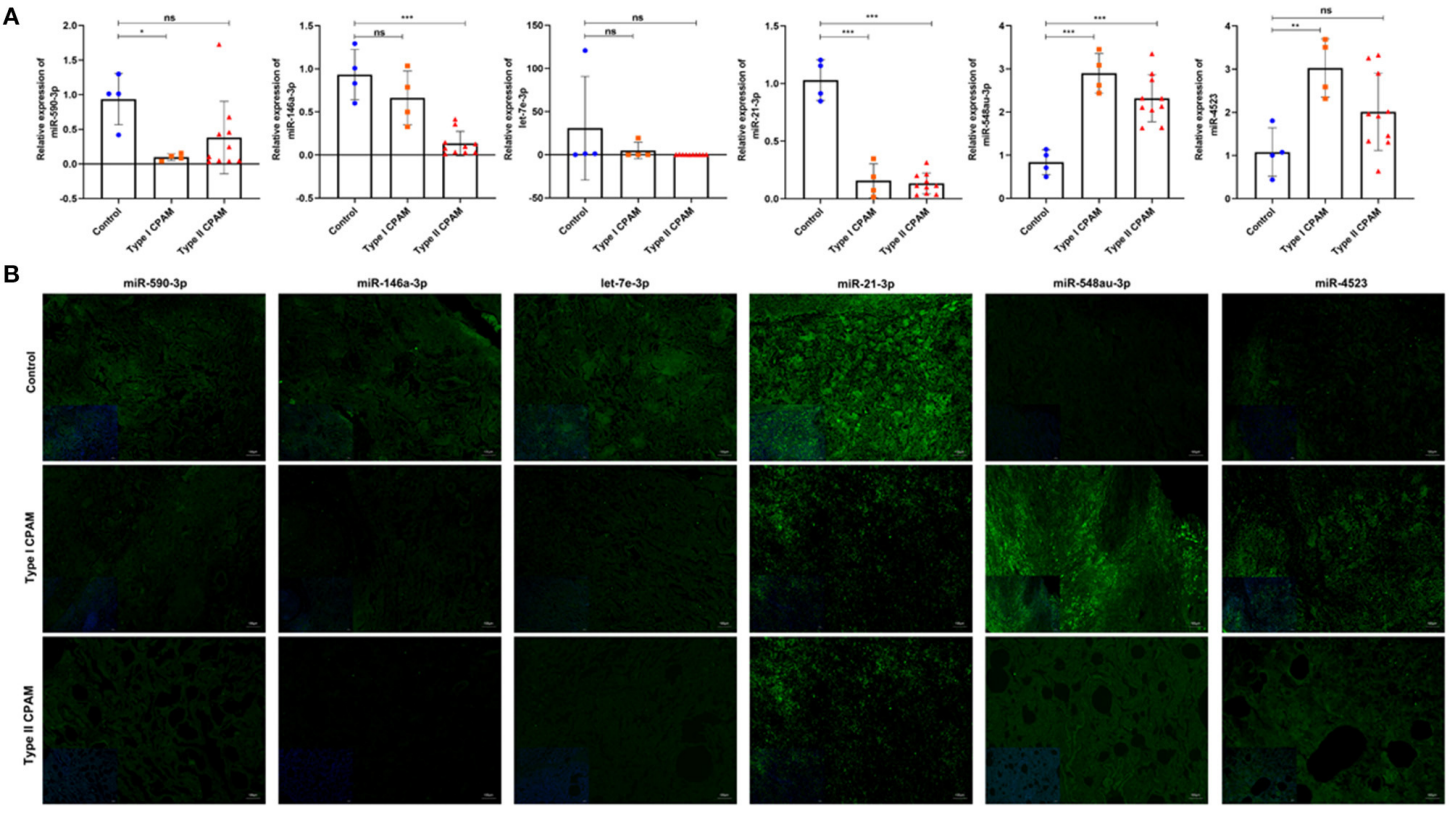
Expression of miRNAs was detected using qRT-PCR and FISH in type I, type II CPAM, and control groups. **(A)** Expression of miR-140-5p, miR-21-3p, miR-590-3p, miR-146a-3p, and let-7e-3p in type I, type II CPAM and control groups, respectively. The data presented as mean ± SD. U6 acts as a control. *P < 0.05; **P < 0.01; ***P < 0.001. **(B)** Expression and distribution of miR-140-5p, miR-21-3p, miR-590-3p, miR-146a-3p, and let-7e-3p in type I, type II CPAM and control groups, respectively. Bar = 50 μm.

To validate the accuracy of the miRNA microarray, the expression of six miRNAs was measured using qRT-PCR in type I and type II CPAM patients, including miR-21-3p, miR-590-3p, miR-146a-3p, and let-7e-3p, which showed downregulation, and miR-4523 and miR-548au-3p, which showed upregulation. As shown in Figure 4A, expression of miR-4523 and miR-548au-3p was significantly increased, whereas expression of miR-21-3p was significantly decreased both in type I and type II CPAM patients. The expression of miR-590-3p and miR-146a-3p was significantly downregulated in type I CPAM and type II CPAM groups compared with the control group, respectively. However, let-7e-3p expression was not changed in both type I and type II CPAM patients compared with the control group. In order to validate the results, the expression and the distribution of these miRNAs were also verified by FISH. The FISH results showed that the green fluorescence of miR-21-3p, miR-140-5p, miR-590-3p, miR-146a-3p, and let-7e-3p was significantly reduced, whereas the green fluorescence of miR-4523 and miR-548au-3p was significantly increased in both type I and type II CPAM patients compared with the control group (Figure 4B). These results were consistent with the results of miRNA microarray, demonstrating the accuracy of the miRNA microarray that was used for further analysis.

### miRNA Target Prediction and Network Construction

**Figure 5:**
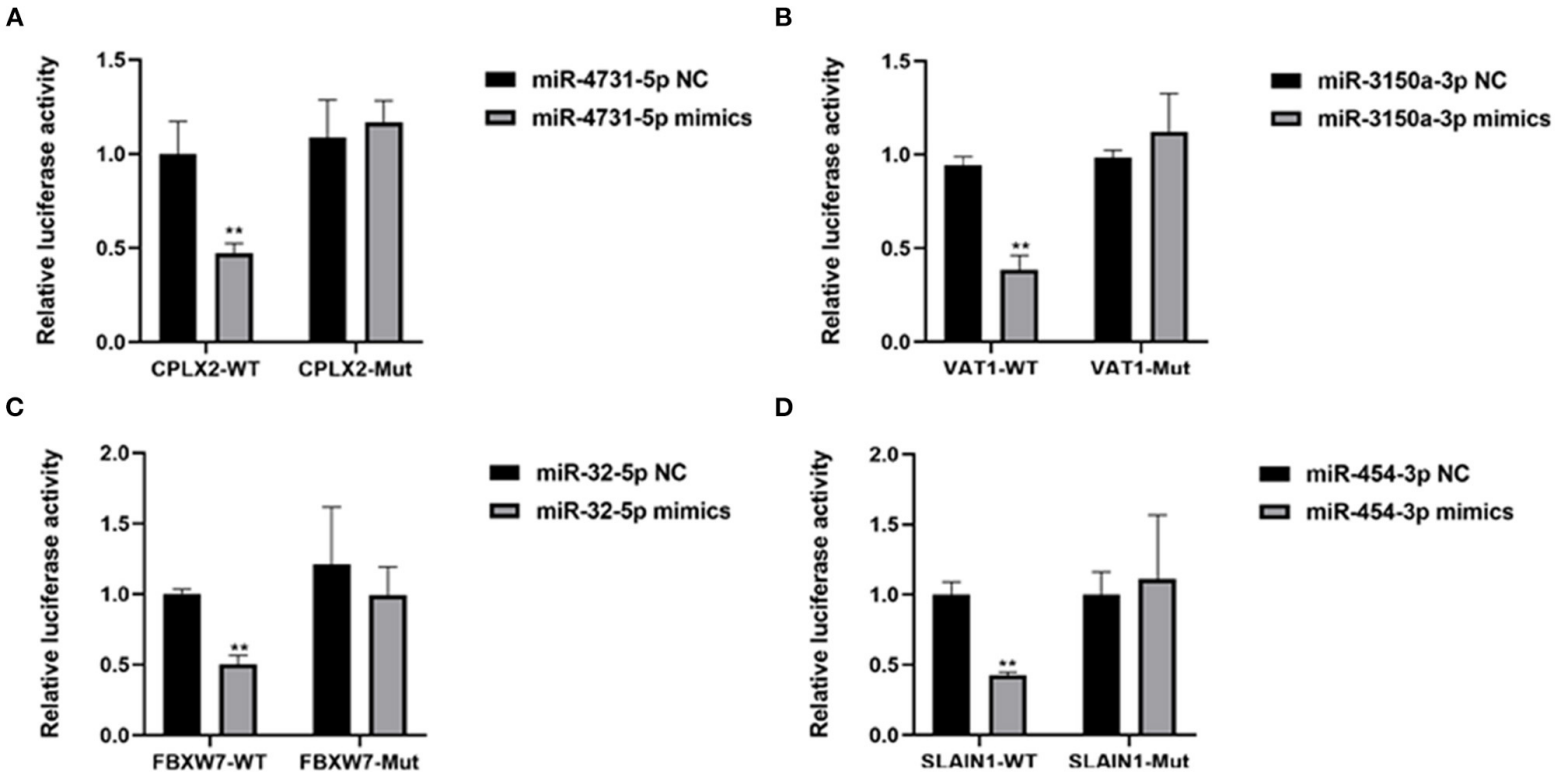
**(A–D)** The regulation of miR-4731-5p to CPLX2, miR-3150a-3p to VAT1, miR-32-5p to FBXW7, and miR-454-3p to SLAIN1 was detected by dual-luciferase reporter assay. **P < 0.01, miRNA mimics group vs. NC group.

It has been demonstrated that miRNA can participate in various development processes by binding the 3′UTR of mRNA. Therefore, we investigated the targets of the common differentially expressed miRNAs by TargetScan7.1 and miRDBV6. For upregulated miRNAs, the results showed that 4,248 and 1,042 targets were predicted for upregulated and downregulated miRNAs both by TargetScan7.1 and miRDBV6, respectively (Supplementary Table 3). According to the miRNA–mRNA network analysis, four miRNAs were the key candidate miRNAs that play important roles in the regulation of CPAM, including miR-4731-5p and miR-3150a-3p, which showed upregulation (Supplementary Figure 1), and miR-32-5p and miR-454-3p, which showed downregulation (Supplementary Figure 2). In addition, targets that showed the best score to bind with these miRNAs were selected, including miR-4731-5p to CPLX2, miR-3150a-3p to VAT1, miR-32-5p to FBXW7, and miR-454-3p to SLAIN1 (Supplementary Figures 1, 2). Dual-luciferase reporter assay showed that the relative luciferase activity was significantly decreased when cotransfection of the 3′ UTR containing wild type binding sequence and miRNA mimics. However, no significant changes were observed when cotransfection the 3′ UTR containing their corresponding mutant binding sequence (Figure 5). These results demonstrated that CPLX2, VAT1, FBXW7, and SLAIN1 were direct targets of miR-4731-5p, miR-3150a-3p, miR-32-5p, and miR-454-3p, respectively.

### GO Analysis Based on Targets of Common Differentially Expressed miRNAs

**Figure 6:**
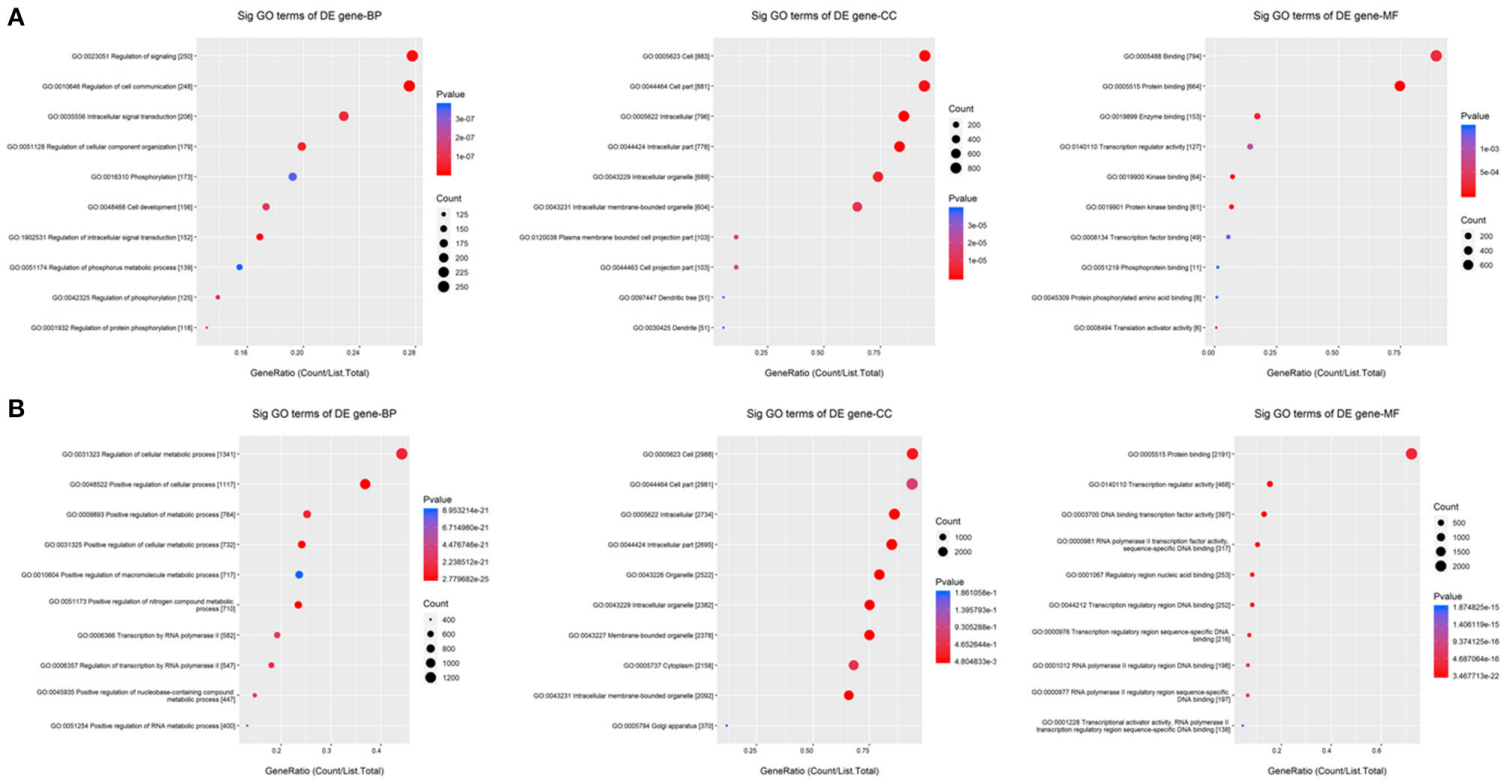
GO enrichment for the targets of common differentially expressed miRNAs. **(A,B)** GO enrichment for the targets of up- and down-regulated common differentially expressed miRNAs, respectively. The ontology covers three domains: biological process, cellular component, and molecular function. Fisher's exact test in Bioconductor's top GO is used to determine whether there is more overlap between the miRNA targets list and the GO annotation list than would be expected by chance. The *p*-value of top GO denotes the significance of GO terms enrichment in the miRNA targets. The lower the *p*-value, the greater the significance of the GO Term.

To determine the function of the miRNAs, GO enrichment analysis was performed using the targets of the common upregulated or downregulated miRNAs. Based on the GOChord plotting function, for biological process (BP), the upregulated targets significantly enriched in regulation of intracellular signal transduction (GO:1902531), regulation of cell communication (GO:0010646), and regulation of signaling (GO:0023051), and the downregulated targets significantly enriched in positive regulation of cellular metabolic process (GO:0031325), positive regulation of nitrogen compound metabolic process (GO:0051173), and positive regulation of cellular process (GO:0048522). For molecular function (MF), upregulated targets were significantly enriched in protein binding (GO:0005515), protein kinase binding (GO:0019901), and kinase binding (GO:0019900), and the downregulated targets were significantly enriched in RNA polymerase II transcription factor activity, sequence-specific DNA binding (GO:0000981), regulatory region nucleic acid binding (GO:0001067), and DNA binding transcription factor activity (GO:0003700). For cellular component (CC), targets with upregulation were significantly enriched in intracellular (GO:0005622), intracellular part (GO:0044424), and cell (GO:0005623), and targets with downregulation were significantly enriched in intracellular part (GO:0044424), intracellular (GO:0005622), and intracellular organelle (GO:0043229) (Figures 6A,B and Supplementary Table 4). These results of GO analysis revealed the function of the targets in CPAM development and progression.

### KEGG Analysis Based on the Targets of the Common Differentially Expressed miRNAs

**Figure 7:**
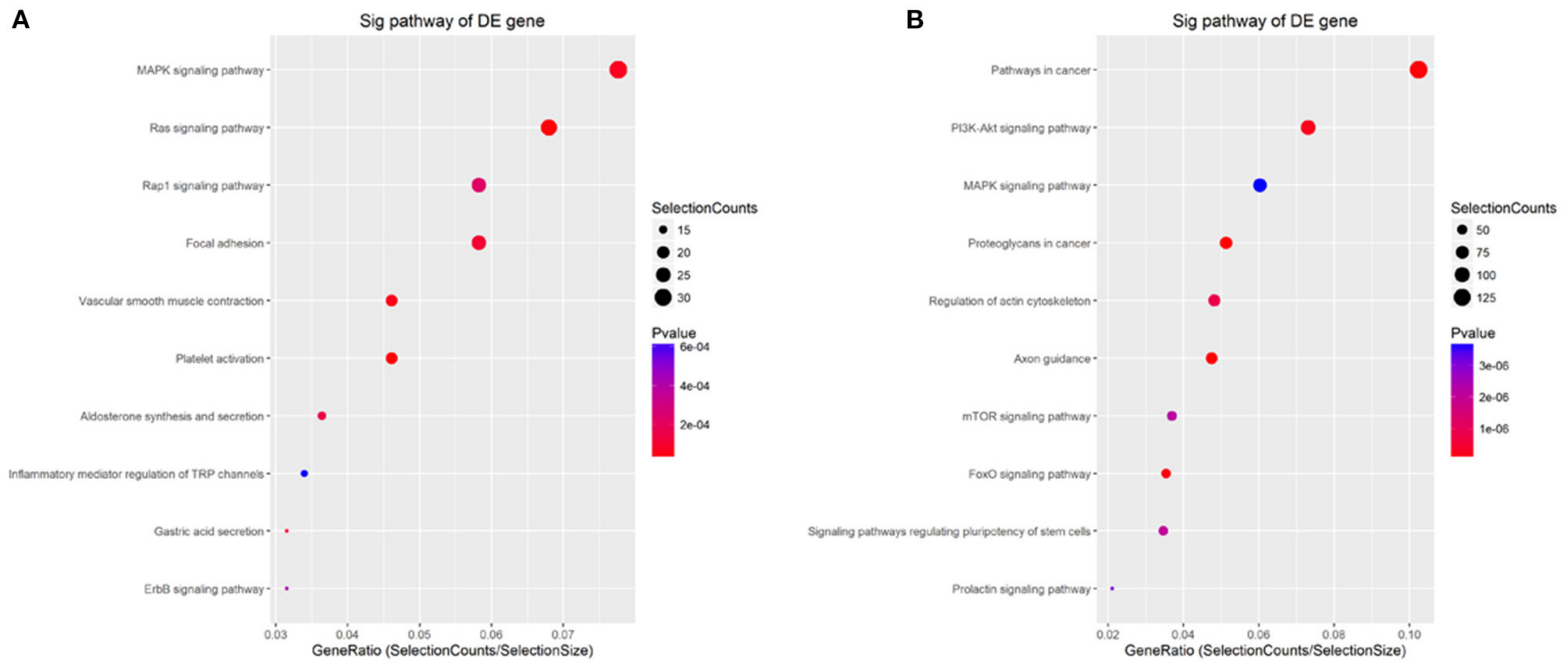
KEGG analysis for the targets of common differentially expressed miRNAs. **(A,B)** KEGG analysis for the targets of up- and down-regulated common differentially expressed miRNAs, respectively. The *p*-value denotes the significance of the pathway correlated to the condition. The lower the *p*-value, the more significant is the pathway. The *P*-value cut-off is 0.05.

KEGG pathway analysis was performed for further analysis of all targets. The results showed that the targets of common upregulated miRNAs mainly were platelet activation (hsa04611, *P* = 1.99e−5), Ras signaling pathway (hsa04014, *P* = 2.56e−5), vascular smooth muscle contraction (hsa04270, *P* = 4.87e−5), MAPK signaling pathway (hsa04010, *P* = 5.88e−5), and focal adhesion (hsa04510, *P* = 9.81e−5) (Figure 7A and Supplementary Table 5). Targets of common downregulated miRNAs mainly were Axon guidance (hsa04360, *P* = 2.14e−5), proteoglycans in cancer (hsa05205, *P* = 3.91e−5), pathways in cancer (hsa05200, *P* = 4.64e−5), FoxO signaling pathway (hsa04068, *P* = 8.40e−5), and PI3K-Akt signaling pathway (hsa04151, *P* = 1.94e−5) (Figure 7B and Supplementary Table 5).

## Discussion

CPAM is a benign, nontumor abnormal lung tissue lesion that leads to perinatal death of the fetus (23). Currently, there is a lack of effective classification biomarkers or methods for CPAM due to its changing types and complicated pathogenesis (4, 24). Therefore, there is an urgent need to investigate diagnostic markers and molecular pathways to regulate the development of CPAM. In this study, differentially expressed miRNAs in type I and type II CPAMs were detected using a miRNA chip, and the feasibility of miRNAs as different CPAM typing markers, as well as the pathways these miRNAs involved in the regulation of CPAM, is elucidated. To our knowledge, this is the first study to establish a potential global miRNA signature in CPAM using high-resolution microarray technology. These results provided a new perspective for the classification of CPAM.

It is of great significance to elucidate the molecular mechanism of CPAM. This will help us correct understanding and provide effective diagnosis methods for CPAM. Much progress has been made in identifying components of the regulatory networks that control lung morphogenesis, including transcription factors, receptors, and miRNAs. miRNAs are endogenous noncoding RNAs with regulatory functions that have shown effects in various lung diseases, such as lung cancer and COPD (25, 26). An increasing number of miRNAs are emerging as potential biomarkers for the diagnosis and treatment of lung diseases. For example, microRNA-155 from sputum might be a noninvasive biomarker for the diagnosis of active pulmonary tuberculosis (27). Furthermore, serum levels of microRNA-92a and microRNA-146a in combination with pulmonary ultrasound scores could predict the severity and prognosis of acute respiratory distress syndrome (28). Microbiota imbalance contributes to COPD exacerbation by increasing IL-17a production *via* miR-122 and miR-30a (29). CPAM is a heterogeneous group of rare lung diseases mainly detected prenatally and characterized by airway dilatation. However, the miRNAs and the underlying molecular mechanisms by which these miRNAs contribute to lung branching are still unknown. In this study, a total of 715 miRNAs were found to be differentially expressed in type I CPAM patients and 106 miRNAs were found to be differentially expressed in type II CPAM patients when compared to healthy individuals. Of them, 14 upregulated and 59 downregulated miRNAs were found in type I and type II CPAM patients. These miRNAs could be the potential biomarkers to distinguish type I and type II CAPM. However, further studies should be performed to select and confirm the core miRNAs.

Generally, miRNA–mRNA networks play a key role in various lung diseases. In CPAM, several genes have been found to play important roles. Hasegawa et al. (30) found that the malignant transformation of CPAMs may be related to the epidermal growth factor receptor (EGFR) pathway, and emphasized the use of EGFR tyrosine kinase inhibitors in epidermal growth factor receptor mutations. Kim et al. (31) found that lack of epithelial PPARγ caused pulmonary airway malformations in mouse fetal lungs. Hong et al. (32) investigated the differential gene expression of type III CPAM and congenital emphysema through gene chip analysis. However, no studies focusing on the miRNA–mRNA networks of CPAM have yet been published. In this study, the miRNA–mRNA networks were established in CPAM. There were 4,248 and 1,042 targets were predicted for upregulated and downregulated miRNAs by TargetScan7.1 and miRDBV6, respectively. The miRNA–mRNA network analysis revealed four key candidate miRNAs that play important roles in the regulation of CPAM, including miR-4731-5p and miR-3150a-3p, which showed upregulation, and miR-32-5p and miR-454-3p, which showed downregulation. In addition, the targets that showed the best scores for binding with these miRNAs were selected, including miR-4731-5p to CPLX2, miR-3150a-3p to VAT1, miR-32-5p to FBXW7, and miR-454-3p to SLAIN1. Of these, miR-32-5p has been shown to inhibit epithelial–mesenchymal transition and metastasis in lung adenocarcinoma by targeting SMAD family 3 (33). Moreover, it also could be regulated by long noncoding RNA PITPNA-AS1 (34). miR-454-3p was found to act as a suppressor in lung cancer (35, 36). FBXW7 has been demonstrated to play a key role in lung cancers regulated by miRNAs (37–39). These results suggested that the miR-32-5p-FBXW7 network contributes to CPAM and needs to be further investigated.

GO is widely recognized as a premier tool for molecular organization and functional annotation. In this study, the upregulated targets for BP were significantly enriched in regulation of intracellular signal transduction, regulation of cell communication, and regulation of signaling, and the downregulated targets were significantly enriched in positive regulation of the cellular metabolic process and nitrogen compound metabolic process. For MF, the upregulated targets were significantly enriched in protein binding and protein kinase binding, and the targets with downregulation were significantly enriched in RNA polymerase II transcription factor activity, sequence-specific DNA binding, and binding to the regulatory region nucleic acid. For CC, both the targets with upregulation and downregulation were significantly enriched in intracellular and intracellular part. These results of GO analysis revealed the function of the targets in ESCC development and progression. These results were consistent with previous studies that these GO items played significant roles in the regulation of different processes in lung development and lesions. KEGG pathway analysis also confirmed these results. This analysis showed that the targets of common upregulated miRNAs were platelet activation, Ras signaling pathway, vascular smooth muscle contraction, and MAPK signaling pathway, and the targets of common downregulated miRNAs mainly were axon guidance, proteoglycans in cancer, pathways in cancer, FoxO signaling pathway, and PI3K-Akt signaling pathway. Of these, Ras, MAPK, and PI3K-Akt signaling pathways have been elucidated in different lung diseases. For example, ERK phosphorylation as a marker of Ras activity has shown prognostic value in non-small cell lung cancer (40), and exposure to mold proteases stimulated mucin production in airway epithelial cells through Ras/Raf1/ERK signal pathway (41). Furthermore, Apelin-36 protects against lipopolysaccharide-induced acute lung injury by inhibiting the ASK1/MAPK signaling pathway (42), whereas fibro growth factor-2 protects against acute lung injury by activating the PI3K/Akt signaling pathway (43). These findings demonstrate that these signaling pathways may contribute to the progression of CPAM, providing a solid foundation for revealing the molecular mechanism of CPAM.

In this study, we identified key miRNAs involved in CPAM development using integrated bioinformatics analysis. However, the relationship between the miRNAs and CPAM progression may be unreliable since the study is based on bioinformatics analysis with a relatively small number of samples. Therefore, in-depth studies with various experimental validations should be required in many clinical samples.

## Conclusion

In summary, 394 upregulated and 321 downregulated miRNAs were found in type I CPAM tissues and 34 upregulated and 72 downregulated miRNA were found in type II CPAM compared with normal tissues. These miRNAs primarily controlled CPAM progression by regulating platelet activation, Ras, MAPK, FoxO, and PI3K-Akt signaling pathways. A miRNA–mRNA network analysis revealed four key miRNAs in CPAM: miR-4731-5p to CPLX2, miR-3150a-3p to VAT1, miR-32-5p to FBXW7, and miR-454-3p to SLAIN1. These findings may provide an important contribution to future investigations aimed at characterizing the role of specific miRNAs in the pathogenesis of CPAM, and contribute to improving diagnosis and treatment.

## Data Availability Statement

The datasets presented in this study can be found in online repositories. The names of the repository/repositories and accession number(s) can be found in the article/Supplementary Material.

## Ethics Statement

All participants signed an informed consent form. All the experiments were approved by the Ethics Committee and the ethics of Guangzhou Women and Children's Medical Center, Guangzhou Medical University.

## Author Contributions

LL conceived and designed the experiments. JZ, WL, JL, JP, FW, and JT performed the experiments. QY, LZ, and DH analyzed the data. LL and JZ wrote the manuscript. All authors read and approved the final manuscript.

## Funding

This work was supported by the Guangzhou Municipal Health and Family Planning Commission, Featured Clinical Technique of Guangzhou (2019TS57).

## Conflict of Interest

The authors declare that the research was conducted in the absence of any commercial or financial relationships that could be construed as a potential conflict of interest.

## Publisher's Note

All claims expressed in this article are solely those of the authors and do not necessarily represent those of their affiliated organizations, or those of the publisher, the editors and the reviewers. Any product that may be evaluated in this article, or claim that may be made by its manufacturer, is not guaranteed or endorsed by the publisher.

## Supplementary Material

Supplementary Figure 1 miRNA–mRNA networks constructed by Cystoscope for upregulated miRNAs and their target networks.

Supplementary Figure 2 miRNA–mRNA networks constructed by Cystoscope for downregulated miRNAs and their target networks.

Supplementary Table 1 miRNA expression profiling data produced using the Agilent 8 × 60 K miRNA-array platform.

Supplementary Table 2 Differentially expressed miRNAs with statistical significance between type I, type II CPAM and control groups.

Supplementary Table 3 The targets of up- and down-regulated miRNAs predicted by targetscan7.1 and mirdbV6.

Supplementary Table 4 GO classification of the targets for up- and downregulated miRNAs.

Supplementary Table 5 KEGG terms of the targets for up- and downregulated miRNAs.
