# Supplementary figures and images for: Analysis of miRNA Profiles and the Regulatory Network in Congenital Pulmonary Airway Malformations
Source: Front Pediatr. 2021 Nov 18;9:671107. doi: 10.3389/fped.2021.671107 (PMC8637626; doi:10.3389/fped.2021.671107)

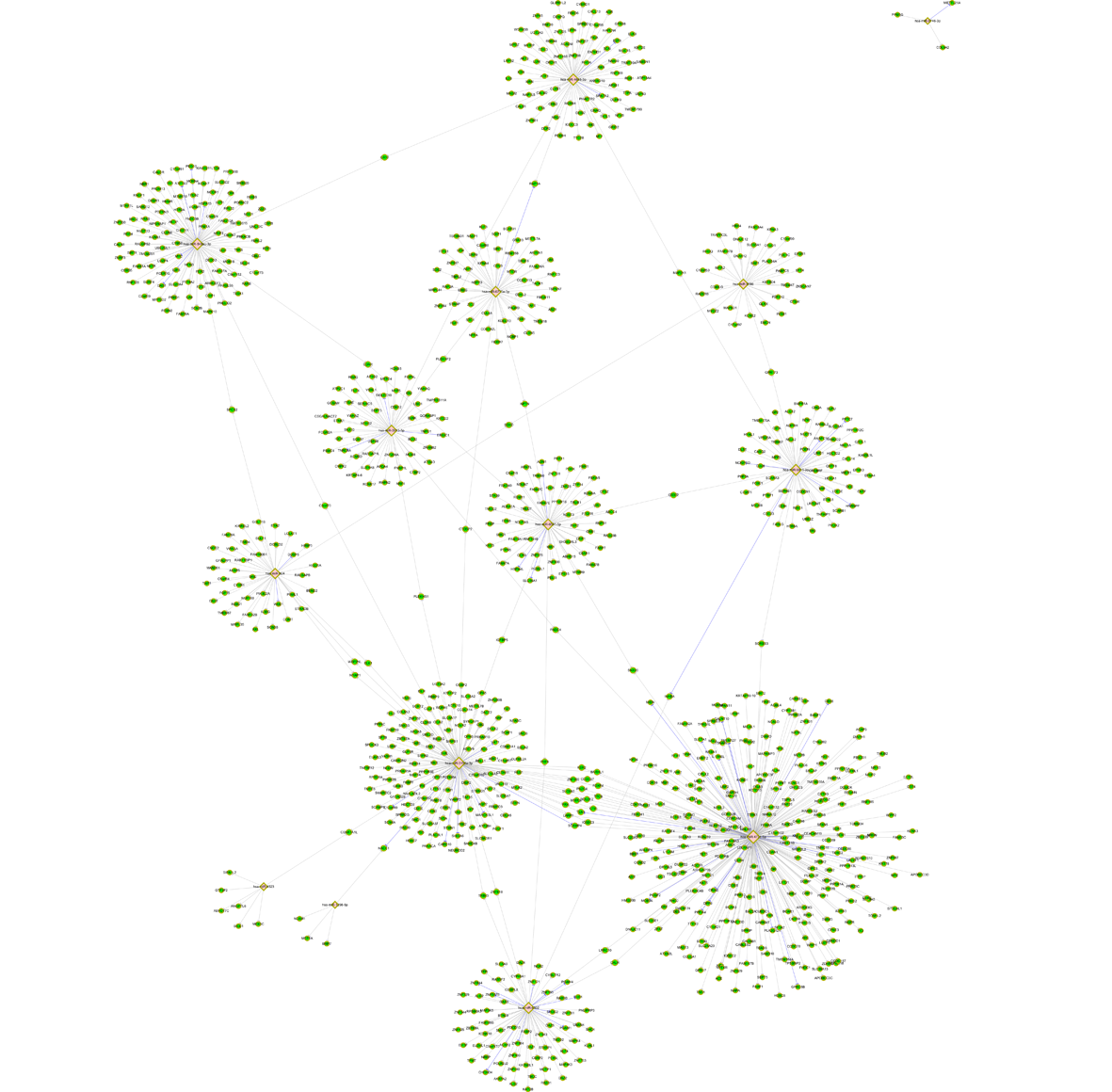

Supplement: Supplementary Figure 1 — miRNA–mRNA networks constructed by Cystoscope for upregulated miRNAs and their target networks. [file Supplementary_Figure_1.TIF]

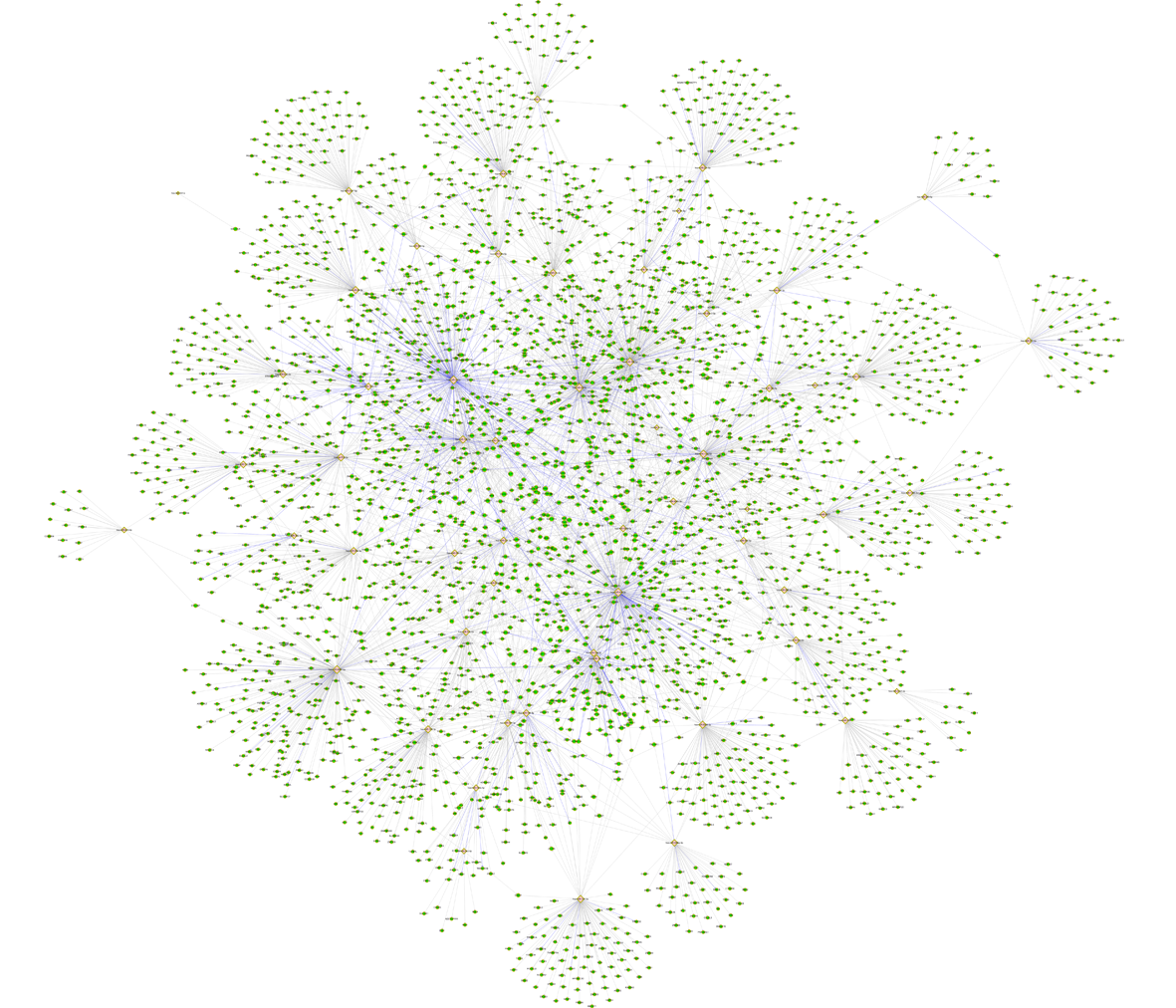

Supplement: Supplementary Figure 2 — miRNA–mRNA networks constructed by Cystoscope for downregulated miRNAs and their target networks. [file Supplementary_Figure_2.TIF]
